# Транскортин: его свойства и функциональная роль в организме человека

**DOI:** 10.14341/probl13482

**Published:** 2024-08-01

**Authors:** А. Шевэ, М. М. Гаджимурадова, Д. Г. Бельцевич, А. Н. Романова, К. Ш. Бегова, Х. В. Багирова, А. К. Эбзеева, Г. А. Мельниченко

**Affiliations:** Национальный медицинский исследовательский центр эндокринологии; Национальный медицинский исследовательский центр эндокринологии; Национальный медицинский исследовательский центр эндокринологии; Национальный медицинский исследовательский центр эндокринологии; Национальный медицинский исследовательский центр эндокринологии; Национальный медицинский исследовательский центр эндокринологии; Национальный медицинский исследовательский центр эндокринологии; Национальный медицинский исследовательский центр эндокринологии

**Keywords:** транскортин, гормоны, связывающие белки, глюкокортикостероиды, буферная система, физиологические механизмы человека

## Abstract

Стероидные гормоны принимают активное участие в целом комплексе физиологических процессов, которые являются основополагающими для нормального развития и функционирования организма. В кровяном русле основная масса стероидных гормонов находится в связанном со специфическими транспортными белками состоянии, в частности с транскортином. Вопросы глюкокортикоидно-белкового комплексообразования при различных состояниях активно изучались во второй половине ХХ века, однако в настоящее время данная проблематика оттеснена на второй план разработкой более точных диагностических методов определения стероидных гормонов. Настоящий обзор литературы представляет накопленные данные о физико-химических свойствах транскортина, генетических факторах, влияющих на его синтез и секрецию. Детально проанализированы опубликованные данные о его физиологическом значении в организме человека в рамках не только гипотезы «свободных гормонов», но и недавно выдвинутой резервуарной гипотезы. Результаты исследований показали, что синтез транскортина был обнаружен в некоторых внепеченочных тканях, в том числе в надпочечниках, однако его роль неизвестна.

## Abstract (en)

Steroid hormones take an active part in a whole complex of physiological processes that are fundamental for the normal development and functioning of the human body. In the bloodstream steroid hormones are bind with specific transport proteins, in particular with transcortin. The matter of changes in hormone-protein complex in various conditions were actively studied in the second half of the twentieth century, but currently this issue has been taken a back seat by the development of high-precision diagnostic methods of steroid hormones determining. This literature review presents accumulated data on the physicochemical properties of transcortin, genetic factors affecting its synthesis and secretion. Published data on its physiological significance in the human body are analyzed in detail within the framework of not only the “free hormone” hypothesis, but also the reservoir hypothesis. Research results have shown that the synthesis of transcortin has been detected in some extrahepatic tissues, including the adrenal glands, however, its role is unknown.

Кортизол-связывающий белок, также известный как транскортин (ТК) или серпин А6, является основным транспортером глюкокортикостероидов (ГКС), в частности кортизола. Около 95% кортизола связано с белками-переносчиками, остальные 5% находятся в свободном или активном состоянии и способны связываться со специфическими рецепторами в клетках. Белки-переносчики кортизола представлены ТК (до 80–90%), обладающим высокой аффинностью, но низкой емкостью и альбумином (до 10%), который, в свою очередь, имеет низкую аффинность и значительно большую буферную емкость [1].

ТК был открыт в 1956 г. двумя независимыми группами ученых, Daughaday et al. и Bush et al., которые продемонстрировали методом равновесного диализа присутствие в плазме крови специфических глобулинов, обладающих очень высоким сродством к кортикостероидам [2][3]. Впоследствии его роль в качестве переносчика была тщательно исследована в равной степени, как и корреляция объема буферной системы и уровня циркулирующего кортизола, а также факторов, оказывающих влияние на его концентрацию и аффинность.

Известно, что стероидогенез, в частности выработка ГКС, есть у всех позвоночных, включая рыб, однако ТК вырабатывается лишь у наземных позвоночных животных и водных млекопитающих. Несмотря на то, что аффинность ТК к ГКС широко варьируется между различными видами животных, основной функцией у всех остается регулирование уровней несвязанных или «свободных» ГКС, циркулирующих в нормальном физиологическом состоянии. Учитывая, что емкость ТК в нормальной плазме человека превышает концентрацию кортикостероидов в состоянии покоя, он действует как буфер, направленный на нивелирование сильных колебаний уровня кортизола, вызванных пульсирующими циркадными ритмами секреции.

## СТРУКТУРА

ТК — это транспортный гликопротеин с массой 50–60 кДа [4]. Человеческий ТК производится гепатоцитами в виде предшественника, который состоит из 405 аминокислотных остатков, включающих 22-аминоксилотных пептида. При его отщеплении образуется зрелый полипептид (содержащий 383 аминокислотных остатка), который циркулирует в концентрации от 30 до 52 пг/мл. Каждая молекула ТК содержит пять активных сайтов N-гликозилирования. Различная 2- и 3-антенарная структура объясняет гетерогенность ТК (размер и заряд). Гликозилирование в Asp238 необходимо для образования третичной структуры белка и создания активного сайта связывания стероидов. Каждая молекула ТК имеет один такой сайт, в котором триптофан в 371 позиции (Trp371) напрямую участвует в связывании стероидов. Многократное увеличение концентрации ТК во время беременности обусловлено гликозилированием в 3-антенарном участке, что приводит к увеличению содержания сиаловых кислот и к уменьшению клиренса сиало-гликопротеиновых рецепторов в печени [5].

По текущим данным базы NCBI Protein отмечается относительно низкая степень сходства последовательностей между ТК разных видов млекопитающих (60% сходства между человеческим и крысиным ТК) по сравнению с ГКС рецептором (91%). Важно отметить, что в зрелом полипептиде ТК человека наиболее плохо сохранившийся участок (участок с низкой степенью сохранности) (49%) соответствует 330–360 аминокислотным остаткам, включающим петлю реакционного центра [6].

## ТРАНСКОРТИН КАК ПРЕДСТАВИТЕЛЬ СЕМЕЙСТВА SERPIN

Считается, что суперсемейство белков SERPIN (сокр. serpine protease inhibitors, «ингибиторы сериновых протеаз») претерпело дивергентную эволюцию более 600 миллионов лет назад и встречается у всех многоклеточных эукариот, причем большинство из данных полипептидов, как следует из названия, ингибирует сериновые протеазы [7]. Все типы SERPIN имеют сходную вторичную структуру, которая включает три β-слоя (листа), 8–9 α-спиралей и домен, содержащий полуконсервативный активный центр. Часть активного центра представлена вариабельным сайтом связывания (важнейший компонент селективности SERPIN) с протеазами. Активные SERPIN существуют в метастабильном состоянии и претерпевают конформационные изменения в более стабильные формы для выполнения различных функций. В результате уникального и обширного конформационного изменения, которое достигается путем отщепления серина, происходит разрушение каталитического центра, что предотвращает высвобождение протеазы из комплекса, а также ведет к ее структурным изменениям [8]. Протеаза, которая теряет свою структурную целостность, подвержена атаке и деградации другими протеазами. Таким образом, большинство SERPIN способствуют более быстрому обороту родственных протеаз и контролируют специфическую протеазную активность [9]. Результатом конформационных изменений является значительное (~10-кратное) снижение аффинности ТК к кортизолу [6][10].

Это суперсемейство участвует в различных физиологических процессах, включая фагоцитоз, коагуляцию, активацию комплемента, фибринолиз, воспаление и др. [8]. У эукариот серпины делятся на две подгруппы: ингибирующие и неингибирующие серпины. В отличие от большинства членов семейства, ТК (SERPINA6) и тироксин-связывающий глобулин (SERPINA7) действуют как неингибирующие молекулы транспорта гормонов [10].

Ген SERPINA6 содержит 5 экзонов, имеет длину в 19-kb и расположен на коротком плече 14 хромосомы (14q32.1) среди нескольких смежных высоко гомологичных генов, которые происходят от одного предкового гена. Промотор SERPINA6 содержит ТАТА-бокс и СААТ-бокс, а также другие элементы высококонсервативной последовательности ДНК, которые, по-видимому, обуславливают его специфическую печеночную экспрессию [11]. Ген экспрессируется в почках, легких, плаценте и поджелудочной железе [4][12].

Уровень общего кортизола у людей на 30–60% является наследственно детерминированным, однако ранее не было идентифицировано никакого конкретного генетического компонента, объясняющего межиндивидуальные различия в уровне утреннего кортизола сыворотки крови. В 2014 г. был организован консорциум CORtisol NETwork (CORNET), целью которого было проведение полногеномного ассоциативного метаанализа (GWAMA). В ходе GWAMA проводился анализ генетических детерминант межиндивидуальных вариаций гипоталамо-гипофизарно-надпочечниковой оси (ГГНО) у 12 597 участников европеоидной расы. Результаты показывают, что <1% дисперсии кортизола в плазме объясняется генетической изменчивостью в одном участке 14 хромосомы. Данный локус охватывает ранее описанный SERPINA6 и SERPINA1, который кодирует α1-антитрипсин, ингибирующий расщепление петли реактивного центра ТК. В пределах данного региона были идентифицированы различия последовательностей ДНК в одинаковых участках хромосом на один нуклеотид, а именно три однонуклеотидных полиморфизма (Single-Nucleotide Polymorphism) или SNP. Дальнейшие исследования в данной области выявили, что эти SNP ассоциированы с изменениями кортизол-связывающей способности ТК, влияют на общие концентрации ТК, а также на иммунореактивность петли активного центра ТК [21][22].

## ФИЗИОЛОГИЧЕСКАЯ РОЛЬ ТК

Первоначально считалось, что роль ТК заключается в транспорте нерастворимого в воде кортизола в сыворотке крови. Однако около 20 лет назад в ходе сравнительного анализа молекулярного поля начали использовать закон действующих масс применительно к гормон-связывающим глобулинам плазмы (ГСГ) (L + R <–> LR), что обычно делается в биомедицинских исследованиях. Закон действующих масс гласит, что количество свободного или связанного гормона в плазме зависит от количества гормона (L), количества связывающего глобулина (R) или сродства одного к другому (двунаправленная стрелка) [13]. В связи с этим возникли разногласия относительно роли ГСГ в регулировании доступа гормонов к тканям.

Существуют три классические гипотезы относительно эффекта ГСГ на доступность гормонов: гипотеза «общего гормона», «свободного гормона» и «связанного гормона». Относительно недавно Malisch и Breuner (2010 г.) представили резервуарную гипотезу, которая является модификацией гипотезы «свободных гормонов» [14].

Согласно гипотезе «общего гормона», присутствующий в плазме гормон свободно поступает в ткани и связывается с рецепторами, следовательно, 100% стероидов плазмы обладают биологической активностью. Стероиды липофильны, поэтому не могут существовать в высоких концентрациях в организме. Таким образом, предполагается, что связывающие глобулины являются простыми транспортными молекулами, функция которых сводится к доставке гормона в ткани по мере прохождения комплекса через капилляры (рис. 1) [15].

**Figure:**
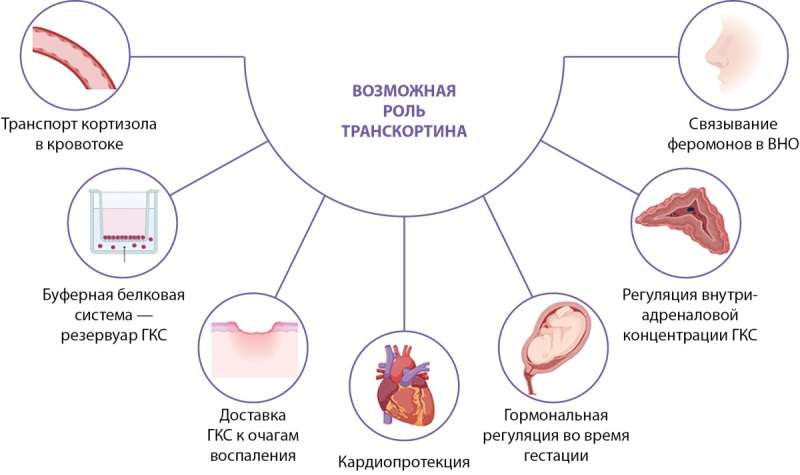
Рисунок 1. Возможная роль транскортина в орагнизме человека.

В свою очередь приверженцы гипотезы «свободных гормонов» утверждают, что только несвязанная (свободная) фракция гормона попадает в ткани и оказывает физиологическое воздействие. ТК обычно имеет размер 50–60 кДа [4]. В нормальных гомеостатических условиях пространства между эндотелиальными клетками, выстилающими стенки капилляров, слишком малы для прохождения больших молекул. ТК, являясь гидрофильной молекулой с большой массой, не может диффундировать через мембраны в клетки, следовательно, кортизол, связанный с ТК, не может проникать в интерстициальную жидкость и остается в плазме. Таким образом, потенциально ограничивается (регулируется) доступ гормонов к тканям. Доказательства в пользу данной гипотезы были продемонстрированы в исследовании in vivo Qian et al., которые сравнивали уровни общего и свободного кортизола в плазме и тканях у мышей в состоянии покоя и при физической нагрузке. По результатам работы изменение уровня кортизола в тканях зеркально отражалось в изменение концентрации свободного кортизола плазмы во времени и сильно отличалось от уровня общего гормона плазмы [24].

Резервуарная гипотеза, как дополнение к вышеуказанной теории, предполагает биологическую активность как свободной, так и связанной фракции гормона [14]. В случае, когда и связывающая способность ТК, и его аффинность уменьшаются, связанный гормон высвобождается и становится биологически активными. ТК плазмы образуют буферную белковую систему, действующую в плазме как резервуар ГКС, которые могут быть непосредственно транспортированы к тканям-мишеням (рис. 1) [16][17]. При различных патологических состояниях, например, лихорадке, сродство ТК к ГКС снижается, в результате чего происходит системное резкое увеличение количества биодоступной фракции гормона [16][18]. В дополнение к его основной роли ТК опосредует адресную доставку ГКС к очагам воспаления (рис. 1) [6][19]. Адресная доставка ГКС, обладающих противовоспалительным эффектом, осуществляется посредством расщепления ТК протеазами, присутствующими в местах воспаления, например, эластазой, высвобождаемой активированными нейтрофилами [6][10]. Аффинность ТК к ГКС снижается в непосредственной близости к сосудистой сети, в результате чего свободная фракция гормона возрастает в 5–10 раз в этом локальном участке [20]. В работе Perogamvros et al. с помощью биоанализа ГКС (исследования показателей клеточного метаболизма in vitro) исследовалась роль ТК в пререцепторном регулировании биодоступности ГКС. Предполагалось, что в случае, когда ТК регулирует доступ ГКС к тканям, изменение уровня ТК должно влиять на активность ГКС на клеточном уровне. Это оценивалось путем погружения клеток HeLa в 10, 20, 50 или 100% сыворотку крови человека, лишенную эндогенного гормона и обогащенную кортизолом. Таким образом, одинаковое количество общего кортизола было доступно вне клеток, однако емкость ТК десятикратно варьировалась. Был зафиксирован гораздо более низкий уровень свободного гормона в 100% сыворотке, что означало: разведение сыворотки приводило к большей доступности свободного кортизола. Такие эффекты разбавления могут быть физиологически значимы при гемодилюции, а также на тканевом уровне при воспалении, сопровождающемся отеком тканей или в синовиальной жидкости, где концентрация ТК определяется тремя процессами: более легким проникновением путем экстравазации, усилением элиминации вследствие расщепления эластазой и дилюцией из-за отека, вызванного воспалением. Эти изменения будут способствовать увеличению концентрации свободного кортизола в местах воспаления, что говорит о достоверности резервуарной теории [23].

## БИОСИНТЕЗ ТК И ЛОКАЛЬНЫЕ ЭФФЕКТЫ В РАЗЛИЧНЫХ ОРГАНАХ

## Синтез ТК в печени

Впервые вывод о синтезе ТК плазмы гепатоцитами сделан Weiser et al., которые продемонстрировали, что гепатоциты крыс синтезируют белок с аналогичным стероидсвязывающим действием и электрофоретическими свойствами, как и ТК [25]. Впоследствии печеночный синтез ТК был подтвержден реакцией трансляции in vitro с использованием мРНК печени, полученной от морских свинок и крыс [26][27]. Предположение, что ТК человека может синтезироваться в печени, было выдвинуто намного ранее (Doe et al. в 1964 г.) и основывалось на том, что концентрация ТК в сыворотке была низкой у больных циррозом печени [28]. Доказательством производства ТК плазмы человека гепатоцитами являются результаты работ Sueda et al. и Khan et al., в которых было показано, что человеческий ТК может быть синтезирован из бесклеточной трансляции печеночной мРНК и клетками HepG2 (клеточная линия гепатомы человека) [29][30].

## Внепеченочный синтез ТК

За последнее десятилетие было проведено несколько ключевых исследований, которые продемонстрировали убедительные доказательства, что ТК может синтезироваться не только в печени, но и в головном мозге, гипофизе, сердце, легких, надпочечниках, яичках, плаценте и др. [31][32][33]. Точная роль ТК в этих тканях до конца не изучена.

В настоящее время изучается непосредственная роль ТК в регуляции действия ГКС, в частности локально в тканях. Guflo et al. сконцентрировались на более детальном изучении надпочечников, одной из наиболее важных структур, обеспечивающих физиологический ответ на стресс. В работе Guflo et al., первоначальной целью которой было выяснение влияния дефицита ТК на секреторно-синтетическую эндокринную активность надпочечников, были выявлены признаки внутриадреналовой продукции ТК. Иммуногистохимический (ИГХ) анализ показал наличие ТК не только внутри клеток клубочковой и пучковой зон, но и в медуллярной зоне [31]. Возможное объяснение состоит в том, что ТК играет роль в регуляции ГКС, влияющих в свою очередь на синтез катехоламинов. Известно, что ГГНС участвует в регуляции синтеза катехоламинов в симпатических ганглиях и мозговом веществе надпочечников за счет индукции различных ферментов. Увеличение АКТГ способствует экспрессии тирозингидроксилазы и допамин- β-гидроксилазы в симпатических ганглиях, но не влияет на их экспрессию в мозговом веществе надпочечников, поскольку MC2R присутствует только в корковом слое надпочечников. В свою очередь в медуллярном слое надпочечников конверсия L-норадреналина в L-адреналин происходит за счет фермента фенилэтоламин-N-метилтранферазы, которая индуцируется кортизолом [34]. В связи с этим ТК можно считать внутриклеточным переносчиком, который способен регулировать внутриадреналовую концентрацию ГКС и опосредованно обеспечивать адекватное реагирование организма на стресс (рис. 1). Это неожиданное открытие требует дальнейших исследований, необходимых для изучения влияния ТК на функцию надпочечников.

Впервые в центральной нервной системе (ЦНС) человека ТК был обнаружен в результате иммуноанализа спинномозговой жидкости в конце 80-х. Наиболее ранние работы исследовали наличие данного белка у других млекопитающих (крыс, морских свинок) [35][36]. В дальнейших исследованиях было обнаружено, что в гипоталамусе человека иммунореактивность ТК была колокализована с классическими нейрогипофизарными пептидами — вазопрессином и окситоцином. Кроме того, ТК был обнаружен в аксональных варикозных расширениях на всем протяжении латеральной области гипоталамуса, перивентрикулярном ядре, во внутренней зоне срединного возвышения, воронке гипоталамуса, а также в нейрогипофизе [37]. Было предположено, что ТК подвергается аксоплазматическому транспорту в крупноклеточных нейронах паравентрикулярного ядра и терминальному высвобождению в портальную или системную циркуляцию вместе с двумя важными стрессовыми пептидами — вазопрессином и окситоцином и тем самым участвует в изменениях ГГНО во время стресса. Длительное время не было достаточных данных о том, обусловлено ли присутствие ТК в нейронах и глиальных клетках его синтезом непосредственно клетками головного мозга или же его наличие было следствием транспорта данного белка из печени. В более поздней работе Sivukhina et al. выявлено, что один и тот же ген (SERPINA6) кодирует ТК как в печени, так и в головном мозге. При секвенировании гипоталамического ТК была выявлена полная его гомология с ТК печени. мРНК ТК, локально вырабатываемая в различных областях головного мозга, исключает теорию транспорта ТК из крови непосредственно в структуры мозга [33].

Наличие внепеченочной экспрессии SERPINA6 на низком уровне было отмечено в легочной ткани человека [12], однако на сегодняшний день ее роль не изучена. В работе Caldwell et al. и Schafer et al. в ходе ИГХ было обнаружено, что реакция «антиген–антитело» к ТК присутствовала почти во всех кардиомиоцитах (цитоплазма, клеточная мембрана). Наиболее выраженная иммунореактивность к ТК обнаружена в волокнах Пуркинье и гладкомышечных клетках артериальной стенки, при этом белок был колокализован с минералокортикоидными рецепторами [32][38]. В ходе полимеразной цепной реакции (ПЦР-тест) мРНК SERPINA6 также была обнаружена в кардиомиоцитах [32]. Авторы отмечают, что обилие ТК в этих клетках указывает на их локальную функциональную важность. Установлено, что экспрессия ТК изменяется при некоторых сердечно-сосудистых заболеваниях. Известная чувствительность кровеносных сосудов к повышенному уровню стероидов связана с такими заболеваниями, как артериальная гипертензия (АГ) и атеросклеротическое поражение кровеносных сосудов. Хронически высокая системная концентрация кортизола связана с увеличением тропонина Т в плазме, что указывает на повреждение кардиомиоцитов. С учетом того, что ТК действует как буфер для ГКС, регулируя количество свободного гормона, было предположено, что кардиопротекция может быть одной из функций ТК (рис. 1) [39]. При высоких концентрациях кортизол связывается с минералокортикоидными рецепторами с аффинностью, равной аффинности альдостерона, что создает чрезмерный окислительный стресс в кардиомиоцитах у пациентов с хронической сердечной недостаточностью (ХСН). Повышенный уровень свободного кортикостерона является дополнительным предиктором риска неблагоприятных сердечно-сосудистых событий у пациентов с ХСН. Более того, уровни циркулирующих стероидов повышаются при различных патологических состояниях, в том числе при инфаркте миокарда (ИМ), при котором данные гормоны являются независимыми предикторами сердечно-сосудистой смерти [40].

У женщин доказательства экспрессии SERPINA6 были продемонстрированы в различных структурах — эндометрии, желтом теле [41]. Misao et al. продемонстрировали наличие мРНК ТК и самого белка в плацентарных клетках. Стероидные гормоны и различные белки в амниотической жидкости человека происходят из децидуальной оболочки и хорионического трофобласта [42][43]. Свободная диффузия стероидов через амнион ограничена. Это представляет из себя один из механизмов защиты эмбриона от нежелательного воздействия биологически активных стероидов (избыток ГКС приводит к задержке внутриутробного развития). В дополнение к этому, у млекопитающих существует две изоформы 11β-гидроксистероиддегидрогеназы (HSD). 11ß-HSD1 взаимопревращает биологически активный кортизол и неактивный кортизон, тогда как 11ß-HSD2 преобразует только кортизол в кортизон. Плацентарный 11ß-HSD необходим для защиты плода от высоких уровней материнских ГКС [44]. Белок 11ßHSD2 локализован в синцитиотрофобластах человека [45]. При ИГХ-исследовании со специфическими антителами к ТК человека Misao et al. также выявили окрашивание синцитиотрофобластов, что позволяет сделать вывод о том, что человеческая плацента также продуцирует ТК, который участвует в гормональной регуляции во время гестации (рис. 1). Кроме того, человеческий амнион, хорион и децидуальная оболочка играют важную роль в возникновении и развитии схваток, в частности, происходит выработка большого количества простагландинов, необходимых для сокращения миометрия [46][47]. ГКС вовлечены в регуляцию продукции простагландинов этими тканями. Challis et al. выявили, что потенциальное значение ГКС состоит в увеличении образования простагландинов посредством стимуляции простангландин-Н-синтазы при одновременном снижении их метаболизма путем ингибирования экспрессии простангландиндегидрогеназы [46]. Таким образом, производство ТК in situ плацентой потенциально может быть одним из ключевых факторов регуляции этого процесса.

Любопытно, что ТК можно также обнаружить в носу млекопитающих. Dölz et al. обнаружили этот белок как в вомероназальном органе (якобсонов орган, ВНО), так и в основной обонятельной системе [48]. ВНО относится к периферическому отделу дополнительной обонятельной системы и играет важную роль в формировании полового поведения путем обнаружения феромонов. ВНО хорошо развит у змей, ящериц и большинства млекопитающих. Считается, что у людей данный орган является временной эмбриональной структурой, однако в незначительной части случаев может быть развит в различной степени и у взрослых людей и представляет собой углубление в носовой полости [49][50][51]. Детальное изучение результатов научных исследований позволило сделать вывод об утрате ВНО своей роли в ходе эволюции и постепенном транзите некоторых его функций в другие ткани организма. Несмотря на то, что в большинстве случаев во взрослом возрасте ВНО не функционирует, восприятие феромонов происходит через основную обонятельную систему. В качестве кандидатов на роль феромонов человека выступило несколько соединений стероидной природы, производных прогестерона, например, андростенон или андростадиенон [52]. В связи с тем, что ВНО почти не содержит ядерных стероидных рецепторов, было предположено, что известные эффекты ГКС в ВНО опосредованы ТК [52][53]. Caldwell et al. выдвинули гипотезу, что ТК, вырабатываемый боуменовыми железами и бокаловидными клетками, секретируется в носовую слизь и, помимо ГКС, может связывать прогестиноподобные феромоны (рис. 1). У крыс, связанный с ГКС или прогестиноподобными феромонами ТК, интернализуется как в сенсорных, так и в несенсорных клетках ВНО. Более того, аксоны, содержащие ТК, были обнаружены и в основной обонятельной луковице [48], в связи с чем было предположено, что ТК транспортирует ГКС или феромоны на всем пути к обонятельной луковице. В свою очередь митральные клетки обонятельной луковицы могут передавать информацию о периферических ГКС или феромонах в лимбическую систему. Поскольку ТК-содержащие отростки в обонятельной луковице часто примыкают к кровеносным сосудам, авторы постулируют, что митральные клетки идеально расположены для опосредованной активации центрального и эндокринного ответа на стресс. Однако на сегодняшний день исследования, демонстрирующие физиологические эффекты предполагаемых стероидных феромонов у людей, не смогли подтвердить заявленные результаты [52].

## ЗАКЛЮЧЕНИЕ

Представленные в обзорной статье данные указывают на важную, но неизученную роль ТК, обеспечивающего синтез и доставку стероидов, и открывают путь к дальнейшим исследованиям.

Измерение ТК может играть практическую роль в оценке активной фракции кортизола, что потенциально может быть полезно в клинической практике, когда уровни ТК или аффинность связывания кортикостероидов могут значительно меняться, однако исследования, посвященные изменениям ТК при различных патологиях надпочечников, весьма ограничены. Кроме этого, получены данные о синтезе ТК непосредственно в надпочечниках, выявлено влияние ТК на экспрессию рецепторов АКТГ на мембране адреналовых клеток. Предполагается, что ТК также влияет на ГГНО во время стресса. Полученные результаты указывают на то, что остается неизученной роль ТК в функционировании надпочечников, синтезе и секреции стероидов. При этом с целью дальнейшего использования полученных данных в практике при анализе результатов необходимо учитывать межвидовую гетерогенность свойств ТК.

Несмотря на то, что был достигнут прогресс в понимании механизмов регуляции уровней ТК в плазме и тканях, которые происходят во время воспаления, на сегодняшний день наши представления о роли и влиянии ТК весьма ограничены, в связи с чем необходимо проведение дополнительных исследований. На данный момент исследования, изучающие механизмы, ответственные за изменения ТК во время воспаления, были выполнены лишь на грызунах и, следовательно, должны быть исследованы у людей. Кроме того, пока остаются неясными роль ТК в процессе восстановления и сроки нормализации уровней ТК в плазме при различных состояниях. Наконец, с растущими доказательствами важности ТК в воспалительных реакциях, негативного влияния высоких уровней ГКС на течение различных сердечно-сосудистых заболеваний, заслуживающими внимания представляются исследования, изучающие потенциал лечения плазменным ТК.

## ДОПОЛНИТЕЛЬНАЯ ИНФОРМАЦИЯ

Источники финансирования. Работа выполнена в рамках государственного задания «Разработка новых технологий диагностики и мониторинга опухолей коры надпочечников с использованием метаболомных и протеомных технологий». Регистрационный номер 123021300098-7

Конфликт интересов. Конфликт интересов отсутствует.

Участие авторов. Все авторы одобрили финальную версию статьи перед публикацией, выразили согласие нести ответственность за все аспекты работы, подразумевающую надлежащее изучение и решение вопросов, связанных с точностью или добросовестностью любой части работы.
